# Cordycepin Regulates the Nrf2/Hmox1 Signaling Pathway to Inhibit Ferroptosis and Improve AKI‐CKD

**DOI:** 10.1002/cbf.70330

**Published:** 2026-10-26

**Authors:** Luming Xi, Mengqi Bai, Junhu Li, Zhibo Zhao, Xiaoshuang Zhou

**Affiliations:** ^1^ Shanxi University of Chinese Medicine Third Clinical College Jinzhong Shanxi China; ^2^ Department of Nephrology Shanxi Provincial People's Hospital Taiyuan Shanxi China; ^3^ Department of Nephrology The Fifth Clinical Medical College of Shanxi Medical University Taiyuan Shanxi China; ^4^ Big Data Center of Kidney Disease Shanxi Provincial People's Hospital Taiyuan Shanxi China; ^5^ Shanxi Provincial Key Laboratory of Kidney Disease Taiyuan Shanxi China; ^6^ Medicinal Basic Research Innovation Center of Chronic Kidney Disease, Ministry of Education Shanxi Medical University Taiyuan Shanxi China

**Keywords:** AKI‐CKD, cordycepin, ferroptosis, Nrf2/Hmox1 pathway

## Abstract

Cordycepin (COR) exerts pharmacological effects in various diseases, yet its role in tissue repair following acute kidney injury (AKI) remains to be fully elucidated. This study aimed to investigate whether COR delays the progression from acute kidney injury to chronic kidney disease (AKI‐CKD) and to explore its underlying mechanisms. Network pharmacology was employed to identify shared targets among ferroptosis, AKI‐CKD, and COR using multiple databases. Gene Ontology (GO) and Kyoto Encyclopedia of Genes and Genomes (KEGG) enrichment analyses were performed, followed by molecular docking validation. In vivo, a renal unilateral ischemia–reperfusion (uIRI) mouse model was established in C57Bl/6N mice (*n* = 20), randomly divided into four groups (*n* = 5 each): Control, CKD model, COR, and CKD + COR. COR (10 mg/kg) or saline was administered intraperitoneally daily for 2 weeks. Renal pathology and fibrosis were assessed via H&E, PAS, and Masson staining; renal function by serum creatinine and proteinuria. In vitro, HK‐2 cells were treated with H_2_O_2_ with or without COR, and cell viability was measured by CCK‐8. The Nrf2 inhibitor ML385 was used to verify pathway involvement. The mRNA and protein levels of GPX4, ACSL4, Nrf2, and Hmox1 were determined by qRT‐PCR and Western blot. Cross‐analysis of network pharmocology identified 42 common targets among 459 COR‐related, 843 AKI‐CKD‐related, and 1866 ferroptosis‐related genes. PPI network analysis suggested that TNF, IL6, and Hmox1 are key targets. Enrichment analyses indicated involvement in iron homeostasis and oxidative stress responses. Molecular docking revealed strong binding of COR to Nrf2 and Hmox1 (–8.5 and –6.7 kcal/mol). In vivo, model mice showed elevated serum creatinine and proteinuria, severe tubular damage, and increased fibrosis, all significantly ameliorated by COR. In vitro, H_2_O_2_ reduced HK‐2 cell viability, downregulated GPX4, Nrf2, and Hmox1, and upregulated ACSL4; COR reversed these effects and improved cell survival. ML385 partially blocked COR's protective and regulatory effects, confirming Nrf2/Hmox1 pathway dependence. Our finding indicated that COR inhibits ferroptosis via activation of the Nrf2/Hmox1 signaling pathway, thereby attenuating renal injury and fibrosis and slowing AKI‐CKD progression.

## Highlights

- Cordycepin inhibits ferroptosis by upregulating GPX4 and downregulating ACSL4 in renal tubular epithelial cells.
- Loss‐of‐function experiments confirm that Nrf2/Hmox1 mediates cordycepin's anti‐ferroptotic and renoprotective effects.
- Cordycepin is a promising ferroptosis‐targeted therapeutic for kidney disease.

**Abbreviations**
ACSL4: acyl‐CoA synthetase long chain family member 4
AKI: acute kidney injury
AKI‐CKD: from acute kidney injury to chronic kidney disease
CKD: chronic kidney disease
GPX4: glutathione peroxidase 4
Hmox1: heme oxygenase‐1
Nrf2: nuclear factor erythroid 2‐related factor 2
qRT‐PCR: quantitative (real‐time) polymerase chain reaction
WB: Western blot

## 1 Introduction

Acute kidney injury (AKI), a common clinical critical syndrome, is characterized by a significant accumulation of nitrogenous metabolites, such as serum creatinine and urea nitrogen, and a dramatic short‐term decline in glomerular filtration rate [1]. According to epidemiologic data, 9.1% of individuals globally have chronic kidney disease (CKD) [2], and AKI is recognized as a significant risk factor that hastens the onset of CKD [3]. Renal tubulointerstitial fibrosis is caused by a combination of mechanisms, including organelle dysfunction, cellular senescence, and a chronic, ongoing inflammatory response, throughout the pathophysiology of the shift from AKI‐CKD [4]. There aren't many specific treatments that can effectively halt the progression of this illness in clinical practice today, except for renal replacement therapy. Therefore, developing novel therapies that can safely and effectively delay the progression from AKI‐CKD is one of the most important problems in the study of renal disease.

Persistent damage to renal tubular epithelial cells is a crucial pathologic characteristic of the development from AKI‐CKD [5]. Recent studies have identified ferroptosis, an iron‐dependent type of regulated cell death, as a key factor in this process [6]. This process is characterized by an imbalance in intracellular lipid peroxide metabolism, which leads to a substantial accumulation of reactive oxygen species (ROS) and ultimately results in ultrastructural damage and mitochondrial dysfunction [7]. The Nrf2/Hmox1 signaling pathway, a basic regulatory system to maintain redox homeostasis, plays a major role in the cellular defense mechanism's capacity to tolerate oxidative stress injury by coordinating the production of multiple cytoprotective genes [8]. As AKI‐CKD gets worse, this pathway is often damaged. For example, Nrf2 signaling weakens or Hmox1 doesn't get activated enough, then cells are more sensitive to oxidative damage [9]. Therefore, by selectively activating the Nrf2/Hmox1 pathway, renal tubular epithelial cells' antioxidant capacity may be increased, potentially providing a unique therapy strategy to avoid iron mortality and halt the progression of AKI‐CKD.

Isolated from Cordyceps sinensis, cordycepin (COR) is a natural nucleoside analog (3′‐deoxyadenosine) with various pharmacological activities, including anti‐inflammatory, antioxidant, antifibrotic, immunomodulatory, and organ‐protective properties [10]. COR can significantly improve renal function indicators and histological changes in AKI models, as well as reduce apoptosis by blocking the NF‐κB signaling pathway, according to earlier research in the field of renal illness [11]. COR has demonstrated promise in slowing the progression of diabetic nephropathy and interstitial fibrosis in mice, demonstrating its multi‐target regulatory capabilities [12, 13]. However, comprehensive and systematic investigations have rarely examined whether COR regulates ferroptosis, a critical stage in the development of AKI‐CKD, and whether this action depends on the activation of the Nrf2/Hmox1 signaling pathway. We hypothesized that COR would enhance renal tubular epithelial cells' capacity to scavenge lipid peroxidation products by activating the core antioxidant pathway, given its well‐established antioxidant properties and protective effects in metabolic stress settings. In the end, this intervention would reduce the progression of AKI‐CKD and avert ferroptosis. Given the foregoing information, the current study employed a methodology that merged computational prediction and experimental validation to systematically elucidate the molecular mechanism by which COR regulates ferroptosis to provide a renoprotective effect.

## 2 Methods

### 2.1 Network Pharmacology Investigates How COR Reduces Ferroptosis in AKI‐CKD

#### 2.1.1 Identification of Ferroptosis Targets Regulated by COR for the Treatment of AKI‐CKD

To predict the pharmacological targets of COR, we first retrieved its chemical structure from the PubChem database (https://pubchem.ncbi.nlm.nih.gov/), and then submitted to the BATMAN database (https://bionet.ncpsb.org/) for target prediction. Additionally, potential targets were identified using the CTD database (https://www.ctdbase.org/), and the GeneCards database (https://www.genecards.org/). All candidate targets from these three datasets were integrated and deduplicated to obtain the comprehensive set of COR‐related targets.

For disease‐related targets, the keywords “AKI‐CKD” and “ferroptosis” were used to search the GeneCards (https://www.genecards.org/) and OMIM (https://www.omim.org/) databases.

Finally, the overlapping targets among the COR action targets, AKI‐CKD‐related genes, and ferroptosis‐associated genes were identified using the Venny online tool (https://bioinfogp.cnb.csic.es/), yielding the potential targets through which COR may inhibit ferroptosis to treat AKI‐CKD.

#### 2.1.2 Building a Protein Interaction (PPI) Network

The common targets were uploaded to the STRING 12.0 database to generate a PPI network, with the species limited to Homo sapiens and a confidence threshold of > 0.9. The network data were exported in TSV format and visualized using Cytoscape 3.9.1.

#### 2.1.3 GO Function and KEGG Pathway Enrichment Analysis Results

To elucidate the biological functions and pathway annotations of the intersecting target genes, the common targets were submitted to the DAVID database (https://david.ncifcrf.gov/) for Gene Ontology (GO) functional enrichment and Kyoto Encyclopedia of Genes and Genomes (KEGG) pathway analyses. A significance threshold of *p* < 0.05 was applied for all enrichment analyses. The top 10 significantly enriched terms in each category, biological process (BP), cellular component (CC), molecular function (MF), and KEGG pathways, were visualized using the bioinformatics online platform (http://www.bioinformatics.com.cn/).

#### 2.1.4 Creating a Network of Components, Targets, and Diseases

Using Cytoscape 3.9.1 software, we produced a “component‐target‐pathway” interaction diagram to show how COR's target‐pathway network delays the progression of AKI to CKD and prevents iron mortality. The nodes in this network indicate COR, the target, and the KEGG pathway, while the edges show the interactions between them.

#### 2.1.5 Molecule Docking

The PDB database (http://www.rcsb.org/) provided the crystal structure files for the target protein, whereas the PubChem database (https://pubchem.ncbi.nlm.nih.gov/) provided the COR molecular structure data in SDF format. Using AutoDockTool, the protein receptor underwent the pretreatment processes of hydrogen atom addition and water molecule removal. AutoDock Vina was then used to finish the molecular docking simulation of the ligand‐receptor. COR was shown to be strongly bound to the important target proteins when the binding energy was less than −5 kJ/mol. PyMOL software was ultimately used to show the molecular docking results in three dimensions.

### 2.2 Grouping and Modeling of Cells

HK‐2 cells were grown in MEM medium supplemented with 10% fetal bovine serum and 100 U/mL penicillin in a 37°C, 5% CO_2_ cell culture incubator. When the cell density was between 80% and 90%, the original media was thrown away. Following a 0.25% ethylenediaminetetraacetic acid trypsin digestion, the cells were washed 3 times with PBS buffer. The cells were prepared for usage once the cell density was adjusted and the cell culture plate was inoculated. The cells were then stabilized and attached to the wall. The H_2_O_2_‐induced cell damage model was used. Cells were divided into the following groups: Control (CON), COR (40 μmol/L), H_2_O_2_ (150 μmol/L), H_2_O_2_ + COR, and H_2_O_2_ + COR + Nrf2 inhibitor (ML385, 1 μmol/L).

### 2.3 Concentration and Duration of the H_2_O_2_‐Induced HK‐2 Cell Damage Model and Screening for COR Drug Concentration

HK‐2 cells in the logarithmic growth phase were seeded into 96‐well plates at 8000 cells/well with six replicates. After 24 h, the medium was replaced with varying concentrations of H_2_O_2_ (0, 50, 100, 150, and 200 μmol/L), and cells were incubated for 24, 48, or 72 h. CCK‐8 reagent (diluted in serum‐free medium) was then added, and absorbance at 450 nm was measured after 1 h to calculate cell viability. Based on these results, H_2_O_2_‐injured HK‐2 cells were treated with graded concentrations of COR (0, 5, 10, 20, 40, 80, and 160 μmol/L) for 72 h. Cell viability was assessed using the CCK‐8 method as described above to determine the effective concentration range and drug safety.

### 2.4 Animal Grouping and Modeling

Male C57BL/6N mice aged 8–12 weeks were used in this study. Renal unilateral ischemia–reperfusion injury (uIRI) was induced by renal pedicle clamping for 45 min, followed by reperfusion. A 14‐day observation period has been reported in previous studies as an appropriate endpoint for evaluating the transition from AKI to CKD and for assessing renal fibrosis [14]. The dosage and administration route of COR used in this study were as previously described [15]. Briefly, COR (10 mg/kg) or an equal volume of 0.9% NaCl solution was administered via intraperitoneal injection daily for 14 consecutive days, starting from the first day of reperfusion. The mice were randomly divided into four groups: Control (sham operation + saline), model (uIRI + saline), COR alone (sham operation + COR, 10 mg/kg), and model + COR (uIRI + COR, 10 mg/kg). We sacrificed the animals at days 14 after uIRI. Kidney tissues were harvested for the following experiments.

### 2.5 Measurement of Serum Creatinine and Proteinuria

Blood and urine samples were collected at the end of the study for the estimation of serum creatinine and proteinuria (Cat. No. C011‐2‐1, C035‐2‐1). All assay kits were purchased from Nanjing Jiancheng Bioengineering Institute. All ELISA and colorimetric assays followed the manufacturer's protocols.

### 2.6 Renal Histopathology

Kidney tissues were fixed in 4% paraformaldehyde at room temperature for 24 h, then dehydrated, embedded in paraffin, and sectioned. Sections were stained with hematoxylin and eosin (H&E), periodic acid–Schiff (PAS), and Masson's trichrome, followed by dehydration and mounting. Histopathological changes were observed under a light microscope, and images were captured for further analysis.

Renal tubular injury was semi‐quantitatively scored as described in Table 1. For tubular injury assessment, 10 glomerular fields were randomly selected from each section under 400× magnification.

**TABLE 1:** Renal tubular injury score.

Score	Severity of injury	Description (percentage of involvement)
0	Normal	No pathological changes
1	Mild	Focal injury, < 25%
2	Moderate	Mild to moderate injury, 25%–50%
3	Severe	Moderate to severe injury, 50%–75%
4	Very severe	Extensive injury, > 75%

For quantitative evaluation of renal fibrosis, stained sections were scanned using a whole‐slide scanner, and images were analyzed with ImageJ software. The collagen‐specific staining channels were separated using the color deconvolution method. The fibrotic area percentage was calculated by threshold segmentation and binarization as the ratio of collagen‐positive area to total tissue area. At least 10 random fields of view per sample were analyzed, and the mean value was used as the final result. All quantitative analyses were performed in a single‐blinded manner.

### 2.7 Western Blotting

After the group intervention, a lysate containing protease and phosphatase inhibitors was used to lyse the cells, and sonication was employed to ensure sufficient fragmentation. The lysates were centrifuged for 10 min at 12,000 rpm, and the supernatant was gathered for protein quantification using the BCA procedure. SDS‐PAGE electrophoresis was performed after the protein samples were denatured by heating them in a metal bath at 100°C for 10 min after being diluted with sample buffer. After electrophoresis, the proteins were transferred to a PVDF membrane and blocked with 5% skim milk for an hour at room temperature. After that, the membrane was coated with the matching primary antibodies, GPX4 (HUABIO, ET1706‐45), ACSL4 (Selleck, F1153), Nrf2 (Proteintech, 80593‐1‐RR), Hmox1 (Zenbio, R24541), and β‐actin (ABclonal Technology, AC050), and it was incubated for a whole night at 4°C. After four 5‐min TBST washes the next day, the membranes were incubated for 1 h at room temperature with the corresponding secondary antibodies. The membranes were developed after washing, and images were obtained using ECL chemiluminescence.

### 2.8 Real‐Time Quantitative PCR

Total RNA was extracted using a column‐based kit and reverse‐transcribed into cDNA. The mRNA expression levels of *GPX4*, *ACSL4*, *Nrf2*, and *Hmox1* were measured by quantitative (Real‐Time) Polymerase Chain Reaction (qRT‐PCR) using the primer sequences listed in Table 2. The PCR reaction was performed under the following two‐step cycling conditions: initial denaturation at 95°C for 30 s, followed by 40 cycles of denaturation at 95°C for 5 s and annealing/extension at 60°C for 30 s.

**TABLE 2:** List of primer sequences.

Primers	Forward (F)	Reverse (R)
GPX4	CCAGTGAGGCAAGACCGAAGT	TGGCTCCTGCTTCCCGAACT
ACSL4	TTTTTGCGAGCTTTCCGAGTG	GCCGACAATAAAGTACGCAAATG
Nrf2	CCTTCAGCAGCATCCTCTCCA	TCTGTGTTGACTGTGGCATCTG
Hmox1	CACAGGGTGACAGAAGAGGCT	TCTGTGAGGGACTCTGGTCTTTG
β‐actin	TCATCACTATTGGCAACGAGCGGT	GTGTTGGCATAGAGGTCTTTACG

### 2.9 Ferric and Ferrous Iron Assay

Ferrous ion concentrations were assessed using the Ferric and Ferrous Ion Assay Kit (Beyotime, S1066). Briefly, approximately 10 mg of tissue was homogenized in 100 μL of lysis buffer on ice under low‐temperature conditions. The homogenate was then centrifuged at approximately 12,000 × *g* at 4°C for 5 min. For ferrous ion (Fe^2+^) detection, 50 μL of the supernatant and 5 μL of decontamination solution were added to each well of a 96‐well plate. The total volume was adjusted to 190 μL with assay buffer. The mixture was incubated at 37°C for 30 min, and the absorbance was measured at 593 nm. Ferrous ion concentrations were calculated according to the standard curve [16].

### 2.10 Statistical Analysis

For group comparisons, unpaired *t*‐test (two groups) or a one‐way ANOVA followed by Tukey correction (many groups) was used for group comparisons. Each experiment was carried out at least 3 times independently, and the results were processed and interpreted using GraphPad Prism 9.0 software. Differences were considered statistically significant at *p* < 0.05.

## 3 Results

### 3.1 Network Pharmacology Prediction and Target Identification of COR in AKI‐CKD and Ferroptosis

A total of 459 potential targets of COR were identified by screening the GeneCards, BATMAN, and CTD databases. Meanwhile, 843 AKI‐CKD‐related targets were obtained by integrating the GeneCards and OMIM databases, and 1866 ferroptosis‐associated targets were retrieved from the GeneCards database. Cross‐analysis of the three target sets revealed 42 overlapping targets, which were visualized using a Venn diagram (Figure 1A).

**FIGURE 1:**
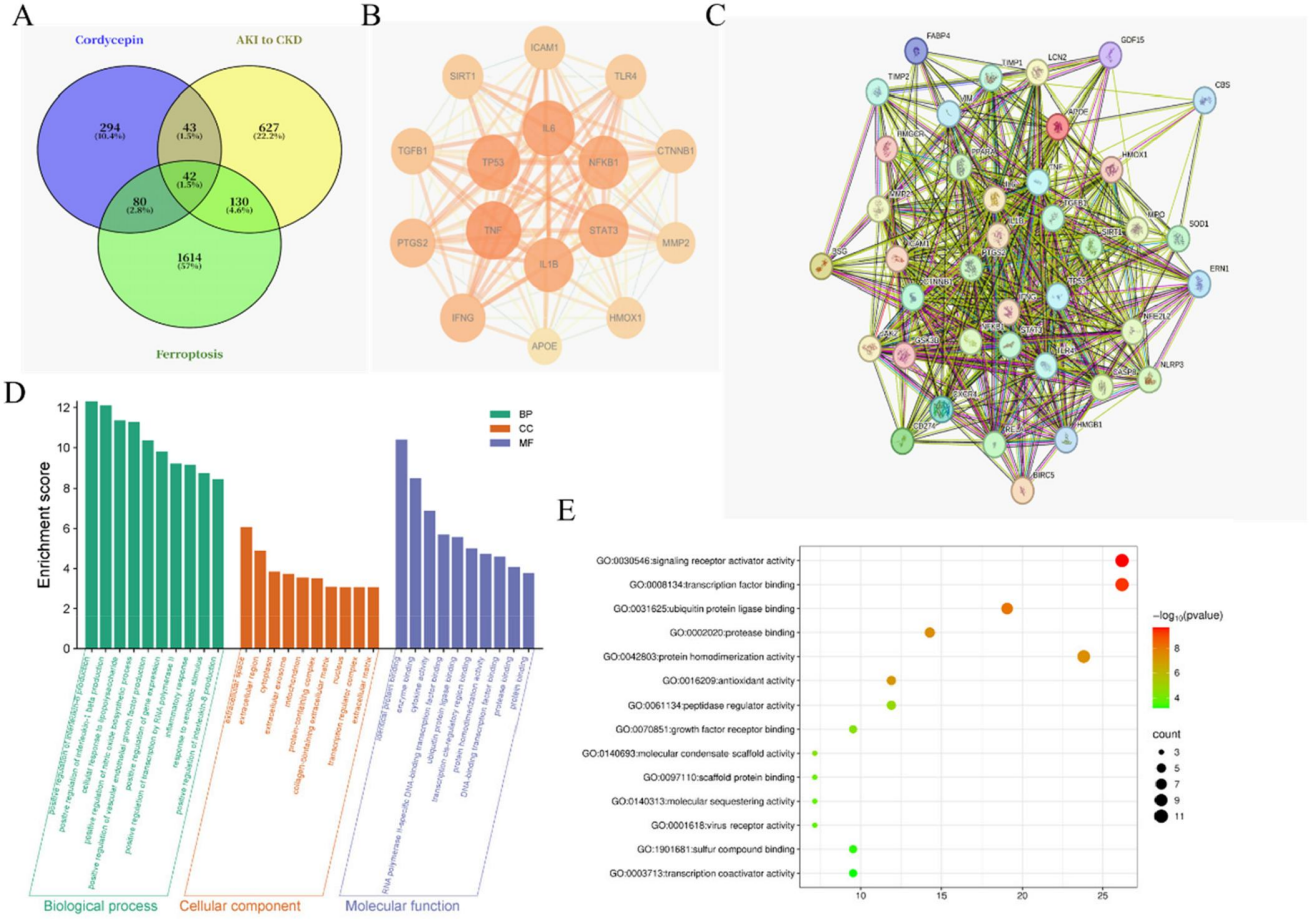
Network pharmacology illustrates the mechanism by which cordycepin alleviates AKI‐CKD through ferroptosis. (A) Venn diagram illustrating the points where ferroptosis, AKI‐CKD, and cordycepin cross. (B, C) 16 important genes, including IL6, TNF, TP53, and Hmox1, were among the core targets selected from the Protein–Protein Interaction (PPI) network (confidence score ≥ 0.9). (D) Biological Process (BP), Molecular Function (MF), and Cellular Component (CC) are among the top 10 entries in the GO functional enrichment analysis. The findings showed that biological processes, including the response to oxidative stress and the control of ferroptosis were the main biological processes where the core targets were enriched. (E) The KEGG pathway enrichment analysis's top 14 pathways with the lowest *p*‐values. The ferroptosis, HIF‐1, and TNF signaling pathways were shown to be the most significantly enriched, according to the results.

The 42 overlapping target proteins were submitted to the STRING 12.0 database with the species set to “Homo sapiens” to construct a protein–protein interaction (PPI) network, aiming to elucidate the molecular mechanisms by which COR regulates ferroptosis and delays AKI‐CKD progression. The resulting network comprised 42 nodes and 480 interaction edges (Figure 1B). In the network, the color intensity of each node reflects its degree value—darker nodes indicate higher connectivity. The PPI network was further visualized and analyzed using Cytoscape 3.9.1. To identify the core targets, the top 16 nodes with the highest degree values were selected as hub genes (Figure 1C).

GO functional enrichment analysis revealed that the common targets were significantly enriched in biological processes (BP) related to inflammation and stress responses, including interleukin‐6 and interleukin‐1β production, positive regulation of nitric oxide biosynthesis, and response to lipopolysaccharide (Figure 1D). In terms of cellular component (CC), the targets were predominantly localized in the extracellular space, exosomes, and mitochondria, suggesting their involvement in mitochondrial function regulation and cellular stress signaling. For molecular function (MF), the targets were primarily enriched in cytokine activity, protein binding, and enzyme binding, supporting the multi‐target regulatory potential of COR. KEGG pathway analysis identified 103 significantly enriched pathways, with the top 14 being associated with transcriptional regulation, ubiquitination modification, and antioxidant responses (Figure 1E).

Collectively, these findings indicate that COR may exert its therapeutic effects in AKI‐CKD through modulation of immune and inflammatory responses.

### 3.2 In Silico Prediction and Molecular Docking of COR Targets in Ferroptosis Regulation

To further prioritize the candidate targets and elucidate the key pathway through which COR may exert its protective effects, we constructed a “component–target–pathway” interaction network and performed molecular docking simulations to predict the binding affinity between COR and the core targets. The findings indicated that core targets of TP53, TNF, IL6, and Hmox1 were closely interconnected (Figure 2A). Notably, Hmox1, a key downstream effector of the Nrf2 pathway, was significantly enriched, suggesting that COR might inhibit ferroptosis through activation of the Nrf2/Hmox1 axis, thereby delaying the progression of renal injury. This provided the theoretical basis for our subsequent mechanistic investigation. Molecular docking further revealed that COR exhibited binding energies of –8.5 and –6.7 kcal/mol to Nrf2 and Hmox1, respectively (Figure 2B,C; Table 2), indicating strong binding affinity to these targets.

**FIGURE 2:**
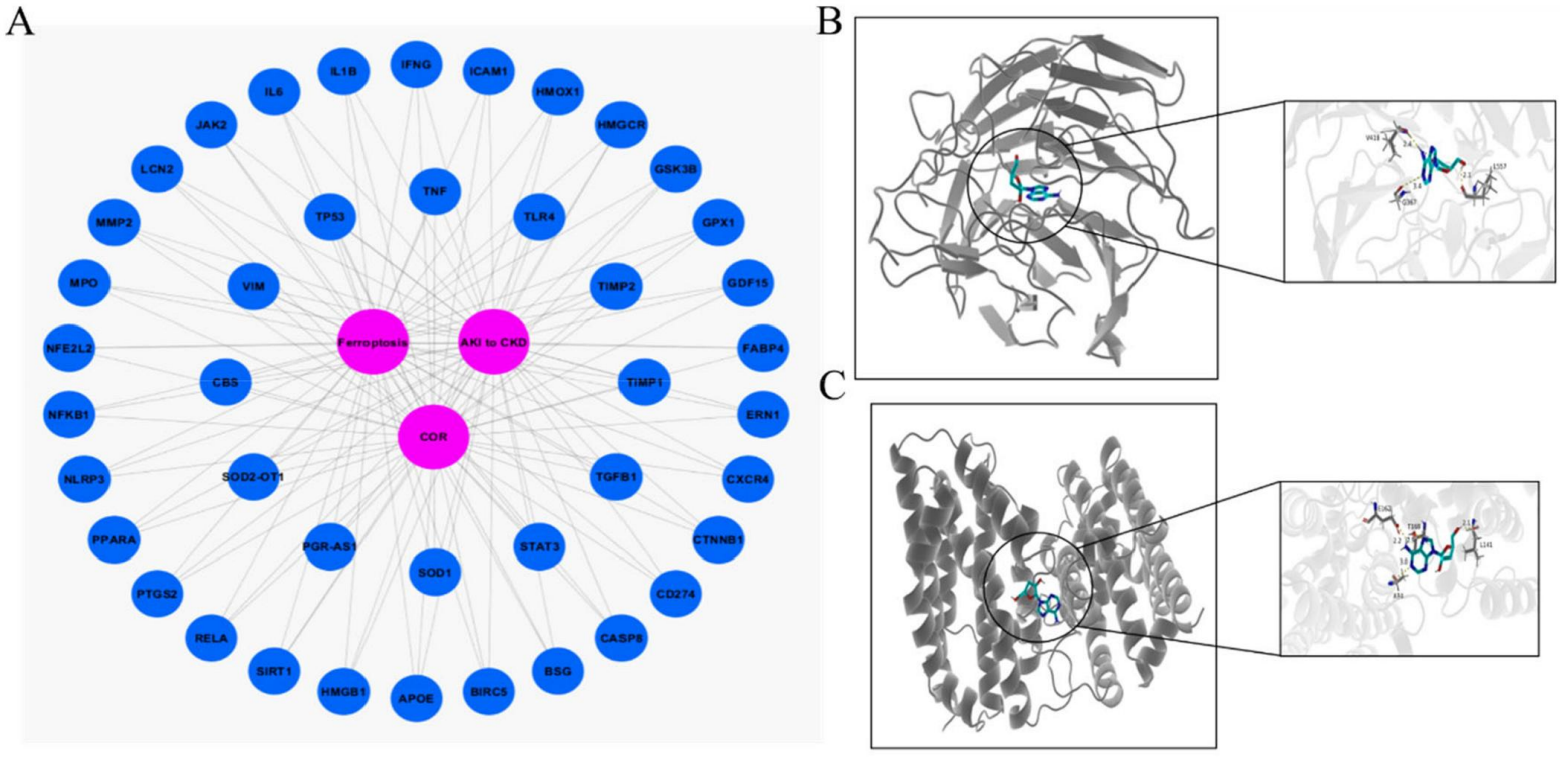
The mechanism by which cordycepin regulates ferroptosis to alleviate AKI‐CKD is revealed via network pharmacology. (A) The “Compound‐Target‐Disease” network of cordycepin provides a visual representation of the possible mechanism via which cordycepin protects the kidneys by controlling the inflammatory signaling axis and iron metabolism. (B, C) Cordycepin has a binding energy of −8.5 kcal/mol with Nrf2 and −6.7 kcal/mol with Hmox1. These findings provide compelling evidence for cordycepin's function as a direct regulator of the ferroptosis pathway by showing that it can bind to important sites within the process with efficiency.

### 3.3 COR Inhibits Ferroptosis via Regulation of the GPX4/ACSL4 Axis In Vivo

Given the robust in silico binding predicted by docking studies, we posed a fundamental scientific question: can this in vitro binding activity translate into in vivo efficacy. To address this, we performed additional pharmacodynamic evaluations in the AKI‐CKD mouse model to validate the in vivo relevance of this interaction and its impact on the pathogenic phenotype.

Serum creatinine and proteinuria levels were elevated in model mice, and COR reduced them (*p* < 0.01) (Figure 3A,B). H&E and PAS staining showed that model mice developed significant tubular injury, including vacuolar degeneration, brush border loss, and cast formation, which were markedly ameliorated by COR (Figure 3C; Table 3). Injury scores were significantly higher in the model group than in the sham group and reduced by COR (*p* < 0.01) (Figure 3D). Masson staining revealed pronounced interstitial fibrosis in the model group, which was effectively prevented by COR (*p* < 0.01) (Figure 3E). QRT‐PCR analysis revealed that the mRNA levels of fibrosis‐related genes, including *Fn1, α‐SMA, Col1a1, Col3a1*, and *TGF‐β*, were significantly elevated in the model group compared with the sham group. In contrast, COR treatment markedly reduced the expression of these genes (Figure 3F–J).

**FIGURE 3:**
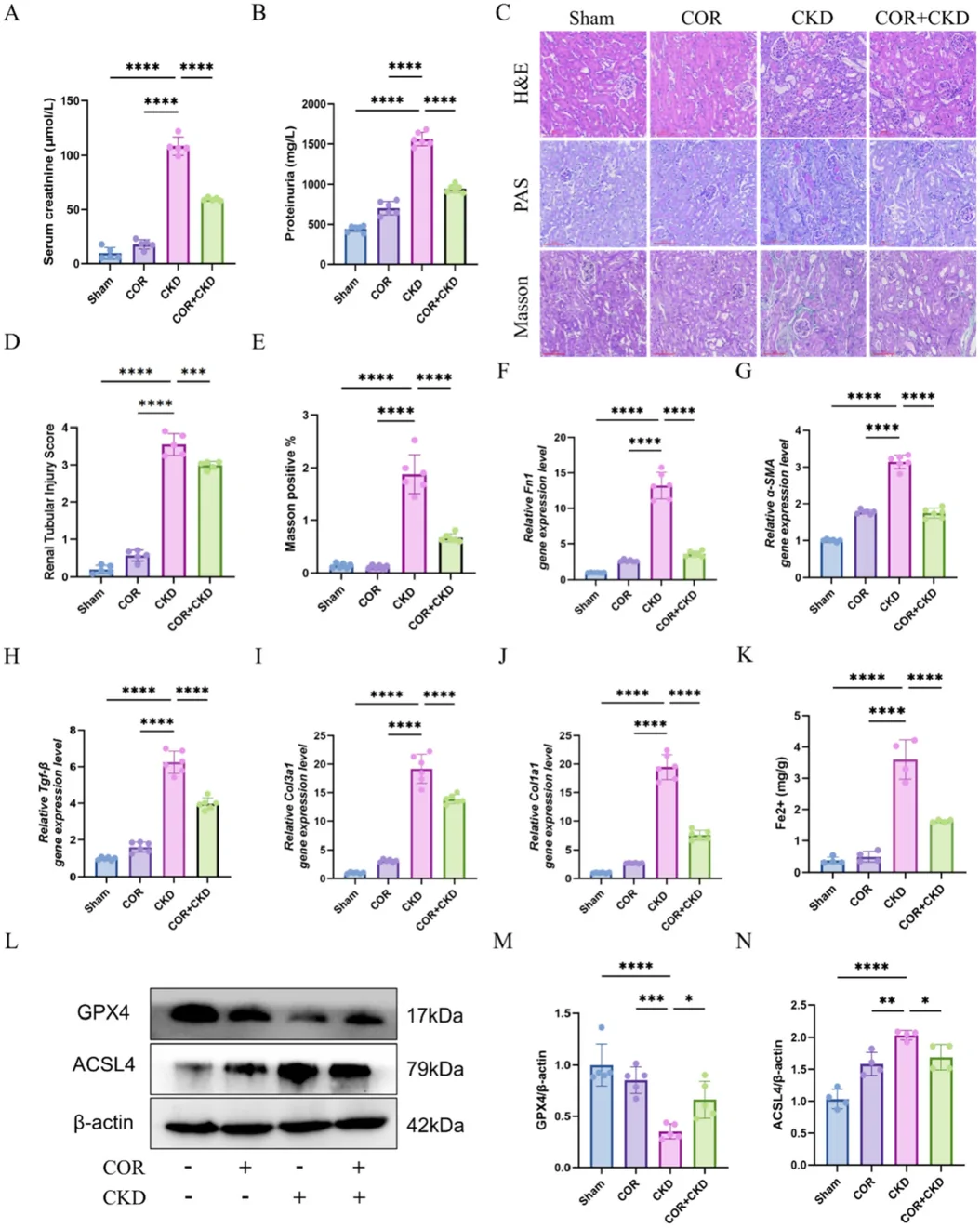
Cordycepin ameliorates renal injury and fibrosis in AKI‐CKD mice by inhibiting ferroptosis. C57BL/6N mice (*n* = 5 per group) were subjected to unilateral renal ischemia‐reperfusion (45 min) to establish an AKI‐CKD model and randomly divided into sham, model, cordycepin (10 mg/kg), and model + cordycepin groups. All treatments were administered for 14 consecutive days. (A, B) Serum biochemical analysis indicated that cordycepin significantly decreased serum creatinine and proteinuria levels (both *p* < 0.01). (C) Representative images of kidney tissue (H&E, PAS, and Masson staining; scale bar = 100 μm) show that cordycepin significantly attenuated tubular dilation, brush border loss, interstitial inflammatory cell infiltration, and collagen deposition. (D, E) Quantitative analysis revealed that cordycepin significantly reduced the tubular injury score and fibrosis area percentage compared with the model group (both *p* < 0.01). (F–J) The fibrosis‐related genes of Fn1, α‐SMA, Col1a1, Col3a1, and TGF‐β were measured by quantitative real‐time PCR. (K) The ferrous iron (Fe^2+^) content in renal tissues was measured by using a commercial iron assay kit. (L–N) Western blot and densitometric analysis demonstrated that cordycepin significantly up‐regulated the expression of the ferroptosis inhibitory protein GPX4 (*p* < 0.05) and down‐regulated the expression of the ferroptosis‐promoting protein ACSL4 (*p* < 0.05), with β‐actin as the internal control. In summary, cordycepin alleviates renal dysfunction during AKI‐CKD progression by modulating the GPX4/ACSL4 protein balance and suppressing the ferroptosis pathway.

**TABLE 3:** Molecular docking binding energies of cordycepin with Nrf2 and Hmox1.

Targets	PDB ID	Molecular binding energy (KJ·mol^−1^)
Nrf2	2FLU	−8.5
Hmox1	1N45	−6.7

Mechanistically, the CKD model group exhibited significantly elevated renal Fe^2+^ levels, a hallmark of ferroptosis, which were markedly reversed by COR treatment (*p* < 0.05) (Figure 3K). Consistently, Western blot analysis revealed that the model group displayed decreased GPX4 expression and increased ACSL4 expression compared with the sham group (both *p* < 0.01), and these alterations were significantly attenuated by COR (*p* < 0.05) (Figure 3L–N).

Collectively, these findings indicate that COR inhibits ferroptosis through regulation of the GPX4/ACSL4 axis.

### 3.4 COR Protects HK‐2 Cells Against H_2_O_2_‐Induced Oxidative Stress

Given the network pharmacology prediction of Nrf2/Hmox1 involvement, we conducted in vitro experiments in HK‐2 cells to verify whether COR protects tubular epithelial cells through this pathway.

To confirm the effects of COR on ferroptosis and the Nrf2 pathway in vitro, we established an oxidative injury model using HK‐2 cells exposed to H_2_O_2_ for 72 h. CCK‐8 assays showed that 150 μmol/L H_2_O_2_ reduced cell viability to approximately 50% (Figure 4A), and this concentration was therefore used in subsequent experiments to ensure model stability. Under this condition, 40 μmol/L COR significantly reversed the H_2_O_2_‐induced reduction in cell viability, indicating a clear protective effect on HK‐2 cells (Figure 4B). We next investigated whether this protective effect is mediated through the Nrf2/Hmox1 pathway and ferroptosis inhibition, and examined the expression changes of ferroptosis markers and Nrf2/Hmox1 pathway components.

**FIGURE 4:**
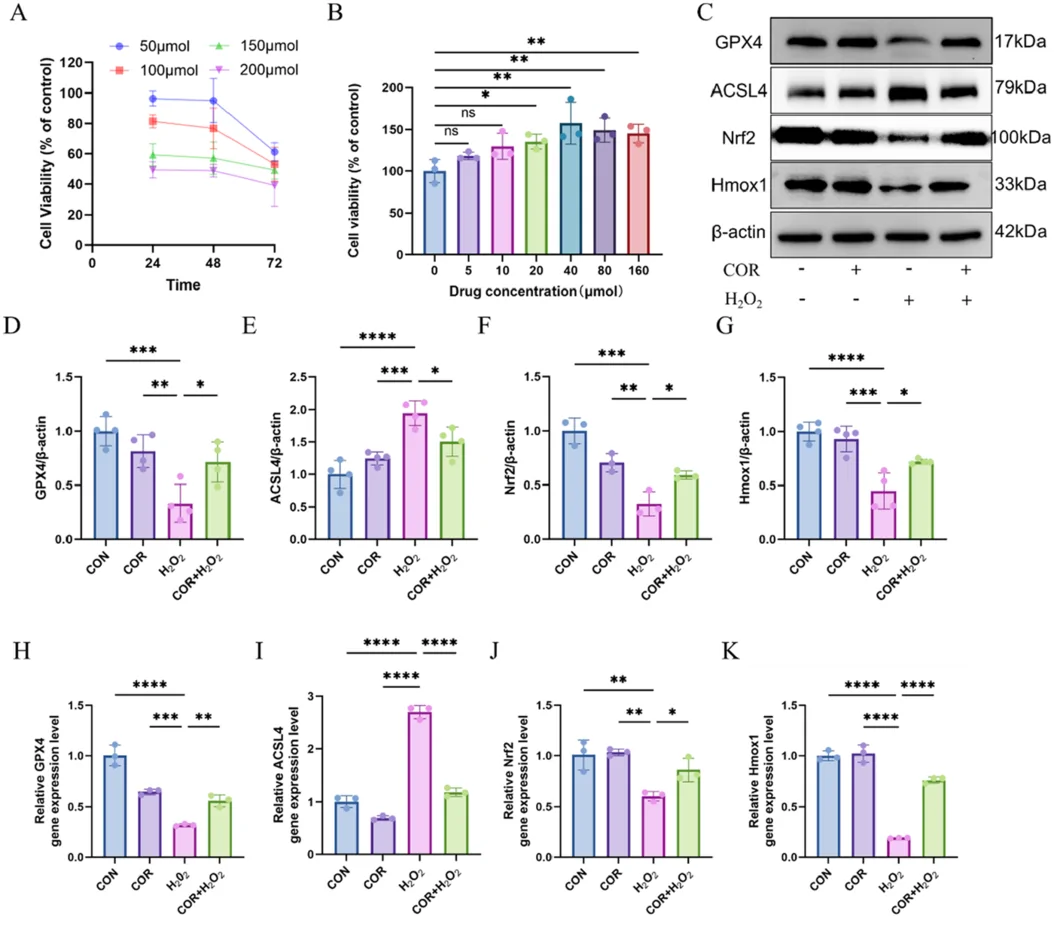
Protective effect of cordycepin against H_2_O_2_‐induced HK‐2 cell injury. (A, B) Screening of 150 μmol/L H_2_O_2_ for 72 h as the optimal injury condition and 40 μmol/L cordycepin (COR, 72 h pre‐treatment) as the effective dose. (C) Western blots show COR upregulates GPX4, Nrf2, and Hmox1 while down‐regulating ACSL4; β‐actin served as loading control. (D–G) Densitometry: vs. the H_2_O_2_ group, GPX4, Nrf2, and Hmox1 increased 1.15‐, 0.8‐, and 0.61‐fold, respectively, whereas ACSL4 decreased 23% (*p* < 0.05). (H–K) qRT‐PCR confirmed parallel mRNA changes: GPX4, Nrf2, and Hmox1 elevated 0.75‐, 0.42‐, and 3.01‐fold, and ACSL4 reduced 56% (*p* < 0.05). Data suggest COR alleviates H_2_O_2_‐induced HK‐2 injury by activating Nrf2/Hmox1 signaling, up‐regulating GPX4, and suppressing ACSL4 to block ferroptosis.

After confirming the protective effect of COR on H_2_O_2_‐treated HK‐2 cells, we further explored the molecular mechanism using Western blot and qRT‐PCR (Figure 4C–K). Compared with the control group, the H_2_O_2_ model group exhibited significantly decreased expression of the anti‐ferroptosis protein GPX4 and markedly increased expression of the pro‐ferroptosis protein ACSL4 (both *p* < 0.01). Notably, both protein and mRNA levels of Nrf2 and its downstream effector Hmox1 were significantly elevated (*p* < 0.01). Following COR treatment, these H_2_O_2_‐induced molecular abnormalities were substantially reversed. Specifically, COR not only upregulated GPX4 and downregulated ACSL4 expression (*p* < 0.05), but also further increased the expression of Nrf2 and Hmox1 at both protein and mRNA levels (*p* < 0.05), effectively counteracting the ferroptotic tendency in HK‐2 cells.

These findings suggest that the cytoprotective effect of COR is primarily attributed to activation of the Nrf2/Hmox1 signaling pathway, which in turn regulates the downstream targets GPX4 and ACSL4 to inhibit ferroptosis in H_2_O_2_‐injured HK‐2 cells.

### 3.5 COR Attenuates H_2_O_2_‐Induced HK‐2 Cell Damage via the Nrf2/Hmox1 Pathway

To determine whether the activation of the Nrf2/Hmox1 pathway is essential for COR's protective effects, we pretreated HK‐2 cells with the Nrf2 inhibitor ML385 prior to COR and H_2_O_2_ exposure. As shown in Figure 5A–I, co‐administration of ML385 significantly reversed the COR‐induced upregulation of Nrf2 and Hmox1 at both protein and mRNA levels (*p* < 0.05), confirming effective suppression of pathway activity. More importantly, ML385 treatment nearly completely abolished the COR‐mediated upregulation of GPX4 and downregulation of ACSL4, restoring their expression to levels comparable to those in the model group (*p* < 0.05). These results unequivocally demonstrate that COR regulates ferroptosis‐related molecules predominantly through Nrf2 pathway activation.

**FIGURE 5:**
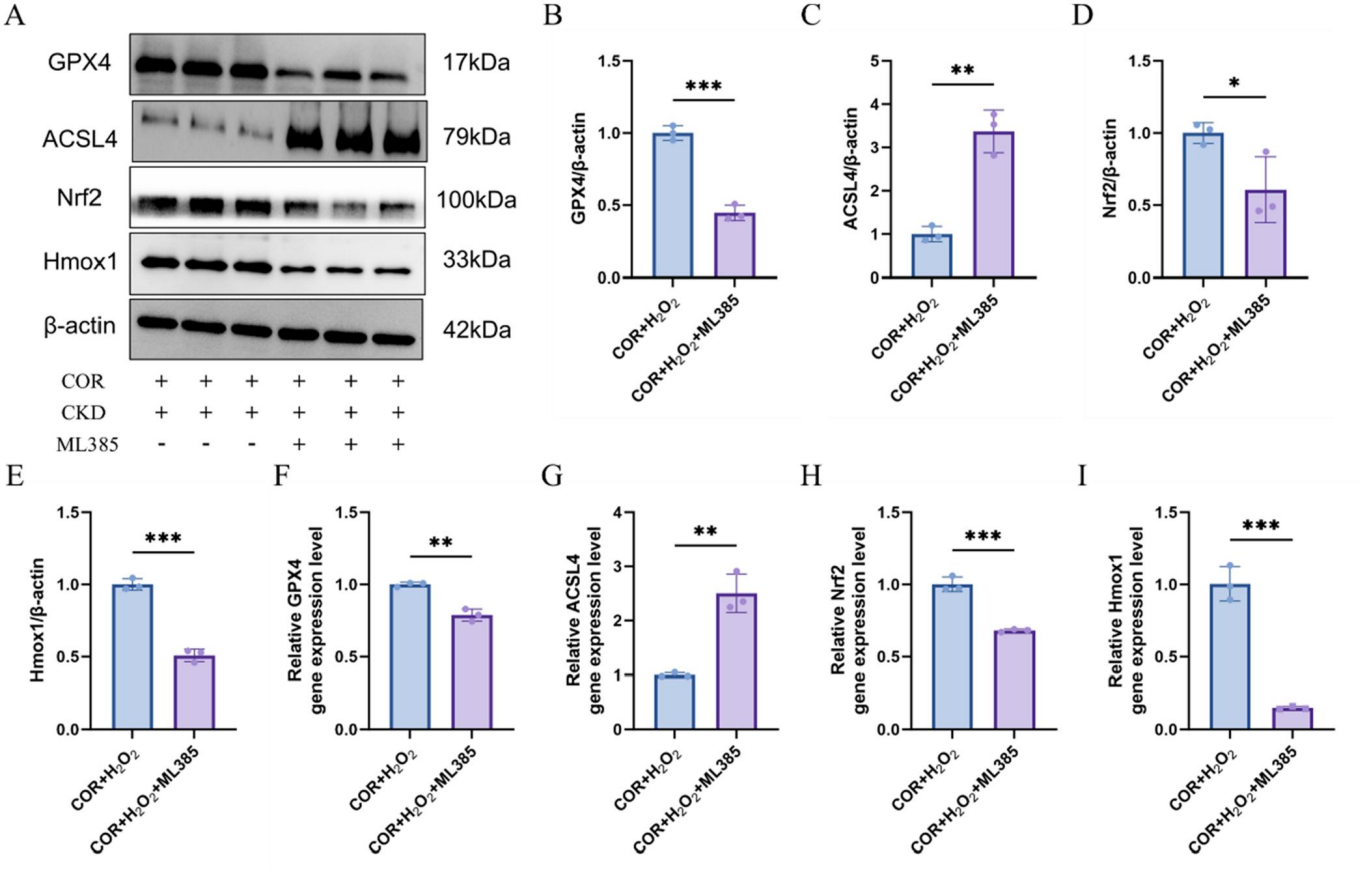
Cordycepin alleviates H_2_O_2_‐induced ferroptosis in HK‐2 cells by activating the Nrf2/Hmox1 pathway. (A) Representative Western blot images. (B–I) Quantitative analysis of protein and gene expression levels (*n* = 3). Cordycepin (40 μmol/L) significantly reversed the H_2_O_2_‐induced dysregulation of GPX4, ACSL4, Nrf2, and Hmox1 (*p* < 0.01). Notably, the Nrf2‐specific inhibitor ML385 (1 μmol/L) completely abolished the regulatory effects of cordycepin, demonstrating the essential role of Nrf2 activation in this process. Conclusion: This experiment confirms that cordycepin relies on the Nrf2/Hmox1 signaling axis to regulate cellular ferroptosis, which is a key mechanism for its cytoprotective effect.

Taken together, our findings provide a comprehensive elucidation of the molecular mechanism by which COR inhibits ferroptosis via activation of the Nrf2/Hmox1 signaling pathway.

## 4 Discussion

AKI is a clinical phenomenon that significantly affects the prognosis of hospitalized patients globally, as its onset significantly raises the prevalence of complications and the risk of mortality [17]. Research has demonstrated a definite causal relationship between AKI and CKD. AKI is both an independent risk factor for the development of CKD and a significant contributor to its progression. A pathophysiological process called the “AKI‐CKD transition” that takes place continually links the two diseases. The two are linked by a chronic pathophysiologic process known as the “AKI‐CKD transition” [18]. It is sometimes difficult to fully recover renal function and tissue structure, and even if some patients survive the acute, potentially deadly phase of this transition, they eventually progress to the chronic phase, which is characterized by interstitial fibrosis [19, 20]. This malignant progression involves complex pathologic mechanisms at multiple systems and levels, mainly involving renal tubular epithelial cell senescence, adaptive repair failure, increasing microvascular thinning, and persistent low‐grade inflammation [21]. These mechanisms contribute to the disease's chronicity by perpetuating a vicious cycle: tissue hypoxia and fibrosis are worsened by the reduction of the microvascular network, microvascular degradation is encouraged by impaired repair capacity, and cellular senescence is accelerated by ongoing activation of the inflammatory microenvironment [22]. In light of this, developing focused therapy strategies that can accurately target the many pathologic systems listed above and effectively stop the disease's progression has become a critical issue in the study of renal disease that requires an immediate solution.

In the current development of therapeutic strategies for renal diseases, research has shifted from managing acute‐phase symptoms exclusively to specifically modulating endogenous renal repair mechanisms to support structural repair and functional recovery of renal tissues [23, 24]. A crucial part of the numerous cellular defense systems is the signaling pathway controlled by the transcription factor NF‐E2 p45‐related factor 2 (also known as nuclear factor erythroid 2‐related factor 2, Nrf2) [25, 26]. Nrf2 is essential for maintaining cellular oxidative stress because it controls the transcriptional output of multiple cytoprotective genes, including phase II detoxification enzymes and antioxidant response elements. Nrf2 is essential for maintaining cellular redox homeostasis because it controls the transcriptional expression of many cytoprotective genes, including phase II detoxification enzymes and antioxidant response elements [27]. Its downstream target gene, heme oxygenase‐1 (Hmox1), is especially notable for its induced expression. It has been demonstrated to have significant anti‐renal fibrosis effects in addition to catalyzing the breakdown of heme to produce active molecules with anti‐inflammatory, antioxidant, and cell signaling regulatory functions, including biliverdin and carbon monoxide [28, 29]. As a selective activator of this route, COR, a naturally occurring small molecule medication, may have therapeutic implications, as demonstrated by our experimental demonstration in this study that it may effectively activate the Nrf2/Hmox1 signaling axis.

COR, a natural nucleoside analog (3′‐deoxyadenosine), was derived from the fungus of the genus Cordyceps and has garnered attention due to its many pharmacological characteristics, which include anti‐oxidative stress, modulation of inflammatory responses, and slowing down of the fibrotic process [30, 31]. Previous preclinical studies have focused on its acute protective benefits in renal ischemia‐reperfusion injury or nephrotoxicity models, and most of these studies have demonstrated that it may significantly lower acute tubular necrosis and oxidative damage [32]. Mechanistically, recent evidence has revealed that COR activates KLF15‐mediated FTH1 transcription by suppressing DNMT1‐dependent promoter methylation, thereby counteracting ferroptotic cell death in renal tubular epithelial cells [33]. Additionally, it has been shown to attenuate renal IRI via inhibition of the p38/JNK signaling pathway, which concurrently suppresses inflammation, apoptosis, and ferroptosis [15]. Concurrently, genetic or pharmacological inhibition of PGK1 has been reported to alleviate AKI by suppressing ALOX12‐mediated ferroptosis [34]. Despite these advances, whether COR can precisely regulate the Nrf2/Hmox1 signaling pathway to avoid iron‐dependent programmed death, or ferroptosis, in renal cells after AKI is a significant scientific question that hasn't been fully addressed yet. Ferroptosis is frequently characterized by abnormal intracellular lipid peroxide accumulation, and Nrf2, a critical hub regulating antioxidant defenses (including glutathione synthesis) and iron metabolism balance, may play a role in this process. Given this, we proposed the following hypothesis: COR enhances renal antioxidant capacity and restores iron homeostasis via activating the Nrf2/Hmox1 signaling axis. This theory inhibits the pathological progression of AKI‐CKD, specifically by preventing ferroptosis. The present study set out to investigate this idea in a rigorous manner.

The present study focuses on the basic mechanism of renal tubular ferroptosis and shows that COR can activate the Nrf2/Hmox1 pathway to provide renoprotective effects, in accordance with the theoretical framework previously indicated. We integrated experimental validation with computational biology to methodically construct a complete chain of evidence. We first identified the target using network pharmacology and molecular docking; and then verified that COR synchronously improves renal function and regulates the expression of the key ferroptosis proteins, GPX4 and ACSL4, in both in vivo and ex vivo models; and we verified the role of Nrf2 activation in tubular ferroptosis in loss‐of‐function experiments using the Nrf2 inhibitor, ML385. Nrf2 activation is necessary for COR's protective action. In this study, the novel signaling axis of “COR‐Nrf2/Hmox1‐ferroptosis” is first identified. By establishing COR as a potential “signaling axis” that can coordinate the several cellular defense systems and avoid the chronicity of illnesses, this refutes the prevalent notion of COR as an acute protective mechanism. It is positioned as a potential “promoter” that can coordinate several cellular defense systems and prevent the disease from becoming chronic, laying the theoretical foundation for the development of potential therapy strategies to halt the progression of AKI‐CKD.

Despite the mechanistic insights provided by this study, several limitations should be acknowledged. First, while we supplemented our in vivo findings with renal Fe^2+^ measurements, the lack of direct real‐time detection of lipid peroxidation and labile iron dynamics in fresh kidney tissues precludes definitive confirmation of ferroptosis. Second, although the pharmacological inhibitor ML385 was used in vitro, the absence of Nrf2‐knockout animals limits the certainty regarding the obligate role of the Nrf2/Hmox1 pathway in mediating COR's effects in vivo. Third, the use of H_2_O_2_‐treated HK‐2 cells as an in vitro model does not fully recapitulate the complex in vivo microenvironment of ischemic AKI; future studies employing ferroptosis‐specific inducers and primary cells would strengthen the conclusions. Fourth, our investigation focused exclusively on ferroptosis, leaving the interplay between ferroptosis and other cell death pathways (e.g., apoptosis and necrosis) in the AKI‐CKD transition unexplored. Fifth, the exclusive use of male mice raises concerns regarding sex‐dependent differences, warranting future validation in female animals. Lastly, as a preclinical study, comprehensive pharmacokinetic, long‐term efficacy, and toxicity evaluations of COR remain to be performed. Collectively, these limitations highlight the need for future studies employing genetic approaches, more diverse cell death assays, sex‐balanced animal models, and rigorous preclinical safety assessments to facilitate clinical translation.

Overall, by blocking the Nrf2/Hmox1 signaling pathway, which stops renal tubular epithelial cells from dying of iron, COR has a nephroprotective effect on the progression of AKI‐CKD for the first time, as our work showed. In addition to providing a new theoretical basis for the clinical application of COR, this result confirms that controlling ferroptosis is a feasible treatment strategy to delay the progression of renal disease.

## 5 Conclusions

This study is the first to systematically demonstrate that COR activates the Nrf2/Hmox1 pathway, upregulating GPX4 and downregulating ACSL4, thereby inhibiting ferroptosis in renal tubular epithelial cells, a conclusion supported by network pharmacology, in vivo, and in vitro evidence. Loss‐of‐function experiments with ML385 further confirmed that COR's anti‐ferroptotic and renoprotective effects are Nrf2/Hmox1‐dependent. These findings reveal a novel mechanism by which COR slows AKI‐CKD progression and provide a theoretical basis for targeting ferroptosis in kidney disease therapy. COR may thus be developed as a therapeutic agent that modulates the Nrf2/Hmox1 axis to retard renal disease progression.

## Author Contributions

**Luming Xi** and **Mengqi Bai:** in vivo and in vitro experiments, data analysis, writing. **Junhu Li:** methodology, data curation, review, and editing. **Zhibo Zhao:** supervision, funding, data analysis, data curation; original draft writing, review, and editing. **Xiaoshuang Zhou:** supervision, funding, review.

## Ethics Statement

All animal experimental procedures were conducted in accordance with the National Institutes of Health Guide for the Care and Use of Laboratory Animals and other internationally recognized guidelines, and they were approved by the Institutional Animal Care and Use Committee of our institution.

## Conflicts of Interest

The authors declare no conflicts of interest.

## Data Availability

The data that support the findings of this study are available from the corresponding author upon reasonable request.
